# Associations of plasma GFAP and P-tau217 with imaging ATN markers and cognitive decline across Centiloid scales

**DOI:** 10.1016/j.lanwpc.2026.101817

**Published:** 2026-02-20

**Authors:** Lin Huang, Fang Xie, Chu-Chung Huang, Qi Huang, Yi-Hui Guan, Qi-Hao Guo, Feng-Feng Pan

**Affiliations:** a Department of Gerontology, Shanghai Sixth People's Hospital Affiliated to Shanghai Jiao Tong University School of Medicine, No. 600, Yi Shan Road, Shanghai, 200233, China; b Department of Nuclear Medicine & PET Center, Huashan Hospital, Fudan University, Shanghai, 200040, China; c Shanghai Key Laboratory of Brain Functional Genomics (Ministry of Education), Affiliated Mental Health Center (ECNU), School of Psychology and Cognitive Science, East China Normal University, Shanghai, China

**Keywords:** Alzheimer's disease, Plasma biomarkers, P-tau217, GFAP, Amyloid PET, Tau PET, Brain atrophy, Cognitive decline

## Abstract

**Background:**

The Centiloid (CL) value offers a standardized metric for quantifying amyloid-β (Aβ) levels in the brain. We aimed to investigate the associations of plasma phosphorylated tau 217 (P-tau217) and glial fibrillary acidic protein (GFAP) with Aβ (A) deposition, Tau (T) accumulation, cortical atrophy (N), and cognitive decline across varying CL scales.

**Methods:**

This study involved 1346 participants who underwent [18F]florbetapir PET, plasma P-tau217 and GFAP measurements, structural MRI (sMRI), and cognitive assessments. A subset of 604 participants additionally completed [18F]MK6240 PET. CL values were stratified into three scales: CL ≤ 10, 10 < CL ≤ 30, and CL > 30. ROC analyses assessed the discriminative abilities of plasma P-tau217 and GFAP across various CL scales. Adjusted regression models examined their associations with Aβ/Tau burden, cortical atrophy, and cognitive decline among different CL scales.

**Findings:**

Plasma levels of P-tau217 and GFAP exhibited a progressive increase across the groups of CL ≤ 10, 10 < CL ≤ 30, and CL > 30 (P < 0.0001), and were most positively associated with CL values within the 10 < CL ≤ 30 range (β = 0.236, P = 0.016; β = 0.206, P = 0.027, respectively). Plasma P-tau217 effectively differentiated between CL > 30 and CL ≤ 30 in cognitively normal (CN) and mild cognitive impairment (MCI) participants (AUC = 0.919 and 0.926, respectively), whereas in dementia participants, it more effectively separated CL > 10 from CL ≤ 10 (AUC = 0.959). A sequential mediation model indicated that CL values influenced the MK6240-SUVR (temporal-meta-ROI) through plasma GFAP, followed by P-tau217, with the most significant effects observed within the 10 < CL ≤ 30 range. Elevated GFAP levels were correlated with reduced cortical thickness and poorer cognitive performance in the CL ≤ 10 group, while increased P-tau217 levels were associated with atrophy and non-executive cognitive deficits in the CL > 10 group.

**Interpretation:**

Plasma P-tau217 and GFAP track early Aβ accumulation, downstream Tau pathology, neurodegeneration, and cognitive deterioration across different CL scales. These biomarkers may provide valuable information for risk stratification and therapeutic targeting of AD within specific CL contexts.

**Funding:**

10.13039/501100001809National Natural Science Foundation of China (Grant No. 82171198, 82501892), Shanghai Municipal Commission of Health Research Project (Grant No. 202440009, 202440010), Shanghai Municipal Science Technology Major Project (Grant No. 2018SHZDZX01), STI2030-Major Projects (Grant No. 2022ZD0213800), and Shanghai Medical Innovation and Development Foundation “Brain Health Youth Fund–Precision Diagnosis and Treatment Research on Alzheimer's Disease” (Grant No. SMIDF-150-2025A30).

**Research in context**

**Evidence before this study**

We searched PubMed for English articles on the links between plasma biomarkers, amyloid-β (Aβ) and tau pathology, brain atrophy, and cognitive decline up to September 1, 2025. We used terms like “Amyloid PET”, “Centiloid”, “plasma biomarkers”, “Tau PET”, “brain atrophy”, “cognitive decline”, and “Alzheimer's disease”. The evidence showed that plasma phosphorylated tau 217 (P-tau217) and glial fibrillary acidic protein (GFAP) accurately predict amyloid status, tau progression, neurodegeneration, and cognitive decline. Centiloid values below 10 exclude neuritic amyloid load, values above 30 confirm it, and values between 10 and 30 indicate a transition from sparse to moderate plaques. However, studies rarely reported plasma biomarker trajectories across the Centiloid scales or their ability to differentiate within this framework, nor their association strength with tau pathology, brain atrophy, and cognitive decline across the Centiloid spectrum.

**Added value of this study**

This study delineates the dynamic trajectories of plasma P-tau217 and GFAP across Centiloid scales, highlighting their significant associations with Centiloid values and the pronounced sequential mediation effects on the relationship between Aβ burden and tau pathology within the 10 < Centiloid ≤ 30 range. Additionally, our findings indicate that plasma P-tau217 is effective in detecting individuals with Centiloid > 30 among cognitively normal and mild cognitive impairment participants, while it more effectively identifying individuals with Centiloid > 10 in dementia participants. Furthermore, our results reveal that elevated plasma GFAP levels are significantly correlated with cortical atrophy and cognitive decline in the Centiloid ≤ 10 group, while plasma P-tau217 is associated with atrophy and non-executive cognitive deficits in the Centiloid > 10 group.

**Implications of all the available evidence**

Our findings underscore the critical importance of monitoring plasma P-tau217 and GFAP during the early stages of Aβ accumulation. When considering the variations of MK6240-SUVR across Centiloid scales observed in our study, we propose that plasma P-tau217 may provide enhanced accuracy in identifying Aβ deposition alongside concurrent tau pathology. Additionally, a lower Centiloid threshold for Aβ positivity may be more suitable for individuals with advanced cognitive impairment. Moreover, our results suggest that the previously observed significant impact of plasma GFAP on neurodegeneration and cognitive impairment in AD may be primarily influenced by P-tau217 and the presence of neurofibrillary tangles in the brain.

## Introduction

Extracellular deposition of amyloid-β (Aβ) and intracellular accumulation of neurofibrillary tangles (NFTs) represent the primary pathological changes associated with Alzheimer's Disease (AD).^1^ The amyloid cascade hypothesis posits that the progressive accumulation of Aβ deposits in the brain is critical for the initiation of AD.^2^ At present, Aβ-PET imaging remains the predominant technique for evaluating Aβ deposition in the brain. Beyond visual assessments conducted by highly trained experts,^3^ the Centiloid (CL) value derived from Aβ-PET serves as a key method for the standardized quantification of Aβ in the brain.^4^ Moreover, it plays a crucial role in the recruitment of participants for anti-Aβ therapeutic interventions.^5^^,^^6^ Recent research consensus suggests that a CL value below approximately 10 effectively excludes the presence of neuritic amyloid load, whereas values exceeding approximately 30 reliably confirm the presence of neuritic amyloid plaques in the brain. The range between approximately 10 and 30 is proposed to represent an intermediate category, indicating a transition from sparse to moderate neuritic plaque presence.^7^^,^^8^

Blood-based biomarkers related to AD, particularly phosphorylated Tau at threonine 217 (P-tau217) and glial fibrillary acidic protein (GFAP), have shown increasing promise for clinical implementation.^9^^,^^10^ Studies have demonstrated that blood levels of P-tau217 and GFAP are closely correlated with cerebral Aβ deposition, both exhibiting high accuracy in identifying Aβ positivity (Aβ+).^11^, ^12^, ^13^ Even prior to the manifestation of clear Tau pathology and clinical symptoms, blood levels of P-tau217 and GFAP exhibit significant elevation.^13^^,^^14^ Furthermore, plasma levels of P-tau217 and GFAP are strongly associated with Aβ deposition-induced Tau pathology in the brain. Research has suggested that plasma P-tau217 mediates the relationship between Aβ and Tau.^15^ Similarly, elevated plasma GFAP levels, indicative of astrocytic reactive activation, are critical in the progression from Aβ deposition to Tau tangles characteristic of the AD pattern.^16^ Moreover, elevated baseline levels of plasma P-tau217 and GFAP are found to be significantly associated with brain atrophy and cognitive decline, which indicates that these two plasma biomarkers play predictive roles in brain neurodegeneration and clinical progress over time.^17^, ^18^, ^19^, ^20^, ^21^ Although the use of CL values in the diagnosis, treatment selection, and efficacy assessment of Alzheimer's Disease (AD) becomes increasingly accepted, and the clinical application of blood biomarkers rapidly advances, there remains a paucity of studies analyzing the progression of plasma P-tau217 and GFAP across the aforementioned CL scales and their effectiveness in distinguishing between different CL scales. Additionally, the relationships between blood-based P-tau217 and GFAP and brain Aβ deposition-induced Tau pathology across various CL scales require further integration and elucidation. Further research is also needed to explore the associations of blood-based P-tau217 and GFAP with brain atrophy and cognitive impairment within the framework of varying CL scales.

This study aimed to investigate the evolutionary patterns of plasma P-tau217 and GFAP in relation to CL values and evaluate their accuracy in distinguishing between different CL scales. Additionally, to enhance the practical applicability of plasma GFAP and P-tau217 within the context of different CL scales, we examined their associations with Amyloid/Tau burdens, cortical atrophy, and cognitive decline in diverse CL scales.

## Methods

### Study participants

Participants for this study were recruited from the Chinese Preclinical Alzheimer's Disease Study (CPAS) research cohort.^22^ We enrolled a total of 1346 participants who underwent structural magnetic resonance imaging (sMRI) [18F]florbetapir (AV45) positron emission tomography (PET) scans, and plasma P-tau217 and GFAP measurements. A subset of 604 participants also underwent [18F]MK6240-PET scans. All participants underwent a battery of standardized neuropsychological assessments, and among them, 311 individuals received a subsequent global cognitive evaluation over a follow-up period spanning 12–71 months. Written informed consent was obtained from all participants or their caregivers. The ethics committee of Shanghai Sixth People's Hospital, affiliated with Shanghai Jiao Tong University School of Medicine, reviewed and approved this study (Approval No: 2019-041).

### Cognitive assessments and clinical diagnoses

Participants' global cognitive performance, functional status, and cognitive domains were evaluated, with specific tests detailed in the Supplementary Methods. The diagnosis of mild cognitive impairment (MCI) was based on the neuropsychological criteria established by Jak and Bondi.^23^ Probable AD dementia was diagnosed according to the guidelines provided by the National Institute on Aging-Alzheimer's Association (NIA-AA).^24^ Participants who scored within the normal range and did not meet the criteria for MCI or dementia were classified as cognitively normal (CN).

### sMRI imaging

Brain sMRI images were acquired utilizing a 3.0 T MRI scanner (SIEMENS MAGNETOM Prisma 3.0 T, Siemens, Erlangen, Germany) at the Department of Radiology, Shanghai Sixth People's Hospital. For details on the preprocessing of the MRI images, refer to the Supplementary Methods. Cortical thickness was quantified as the distance from the gray–white matter boundary to the corresponding pial surface, encompassing six predefined bilateral regions of interest (ROIs): the entorhinal cortex, inferior temporal cortex, middle temporal cortex, superior temporal cortex, inferior parietal cortex, and fusiform cortex.

### Amyloid/Tau PET imaging

Both AV45-PET and MK6240-PET scans were conducted using a PET/CT system (Biograph mCT Flow, Siemens, Erlangen, Germany) with established parameters as previously documented.^25^ Comprehensive details regarding image acquisition and data preprocessing for both AV45-PET and MK6240-PET are provided in the Supplementary Methods. Standardized uptake value ratios (SUVRs) for AV45 were derived using Centiloid mask images (voi_cxt_2 mm.nii and voi_WhlCb_2 mm.nii) from the Global Alzheimer's Association Interactive Network (GAAIN). Validation of our method was performed using the standard PIB dataset, which provided a conversion formula to transform [11C]PIB SUVR into Centiloids.^4^ In the Florbetapir Calibration dataset, a linear regression model was developed to relate [18F]florbetapir to [11C]PIB SUVR through direct image comparisons, establishing the conversion between these two SUVR measures. Consequently, the following formula was derived to calculate Centiloids directly from AV45-SUVR: *Centiloids* = 179.64 × *SUVR*_*Florbetapir*−186.95. The temporal meta-ROI tau SUVR was calculated by including meta-temporal regions such as the entorhinal cortex, inferior/middle temporal cortex, fusiform gyrus, parahippocampal gyrus, and amygdala, with the inferior cerebellar gray matter serving as the reference region.

### Measurements of plasma P-tau217 and GFAP

Plasma concentrations of P-tau217 and GFAP were measured using the Light-initiated Chemiluminescence Assay (LiCA®, Chemclin Diagnostics, Beijing, China). This method utilizes antibody-conjugated luminescent and photosensitive microspheres that generate a signal when brought into proximity by antigen binding. Plasma biomarker concentrations, expressed in pg/ml, were determined by fitting the results to a standard curve. Further description of the measurement is provided in the Supplementary Methods.

### Statistical analyses

Categorical variables were represented as frequencies (percentages) and analyzed utilizing the Chi-squared test. Continuous variables were presented as means ± standard deviation (SD), with multi-group comparisons conducted using analysis of variance (ANOVA) followed by post hoc Bonferroni correction. General linear models were used to examine the interaction effects between Centiloid scales and cognitive stages on plasma P-tau217, plasma GFAP, and MK6240-SUVR (temporal meta-ROI) levels. In cases where significant interactions were observed, post-hoc multiple comparison tests were performed on the Centiloid stratified groups across cognitive stages. Prior to analysis, all biomarker variables (plasma P-tau217, GFAP, and MK6240-SUVR) were log-transformed to ensure normality. The area under the receiver operating characteristic curve (AUC) was employed to assess the efficacy of plasma biomarkers in differentiating various scales of CL values. Differences between various AUCs were compared using Delong test. Within the subgroups with various CL scales, linear regression analysis adjusted for gender, age, and *APOE* ε4 genotype was used to assess the evolution of plasma P-tau217, plasma GFAP, and MK6240-SUVR (meta-temporal) in relation to increasing CL values. Dual mediation models adjusted for gender, age, and *APOE* ε4 genotype were conducted using SPSS PROCESS macro (Model 6) to investigate the associations between the plasma and image indicators, in which the CL value was modeled as the associated variable, plasma GFAP and P-tau217 levels were tested as sequential mediators, and the MK6240-SUVR (meta-temporal) as the outcome variable. All paths are reported as standardized regression coefficients. The associations of z-scored plasma P-tau217 and GFAP with cortical thickness, as well as cognitive function, were evaluated via linear regression models adjusted for gender, age, *APOE* ε4 genotype, and education years. Statistical significance was determined using a two-sided P value threshold of <0.05. Data analyses were executed using IBM SPSS Statistics version 26, MedCalc 23.1.6, and GraphPad Prism version 9.0 (GraphPad Software, Inc.).

### Role of the funding source

The funders of the study had no role in study design, data collection, data analysis, data interpretation, or writing of the report.

## Results

### Participant characteristics

The characteristics of the 1346 participants, categorized by clinical diagnosis, are presented in Table 1. The cohort, averaging 66.7 years old (SD = 7.99), had an average education of 11.3 years (SD = 3.79). Among them, 38.6% were male, and 28.6% carried at least one *APOE* ϵ4 allele. The group included 468 cognitively normal (CN) individuals, 551 with mild cognitive impairment (MCI), and 327 with dementia. Plasma levels of GFAP, P-tau217, CL values, and temporal meta-ROI MK6240-SUVR significantly increased from the CN group to the MCI group and then to the dementia group. The percentage of individuals with CL ≤ 10 decreased from 87.6% in the CN group to 72.6% in the MCI group, and to 28.4% in the dementia group. Conversely, those with CL > 30 increased from 6.6% in the CN group to 18.5% in the MCI group, reaching 61.2% in the dementia group. The 10–30 CL subgroup was notably larger in dementia participants compared to CN, but similar between dementia and MCI groups.

**Table 1:** Demographics and key characteristics of participants.

Index	ALL (n = 1346)	CN (n = 468)	MCI (n = 551)	Dementia (n = 327)
Gender [Male, n (%)]	519 (38.6%)	167 (35.7%)	223 (40.5%)	129 (39.4%)
Age (years)	66.7 ± 8.0	64.0 ± 8.1	67.7 ± 7.2^a^	68.7 ± 8.1^b^^,^^c^
Education (years)	11.3 ± 3.8	12.7 ± 3.2	11.2 ± 3.6^a^	9.6 ± 4.1^b^^,^^c^
*APOE* ε4+ [n (%)]	385 (28.6%)	88 (18.8%)	153 (27.8%)^a^	144 (44.0%)^b^^,^^c^
MoCA-B scores	20.8 ± 6.2	25.7 ± 2.6	21.5 ± 4.1^a^	12.5 ± 4.4^b^^,^^c^
ADL scores	21.4 ± 3.7	20.3 ± 1.6	20.9 ± 2.6	23.9 ± 5.9^b^^,^^c^
Plasma P-tau217 (pg/ml)	0.60 ± 0.58	0.35 ± 0.19	0.46 ± 0.34^a^	1.17 ± 0.83^b^^,^^c^
Plasma GFAP (pg/ml)	154.57 ± 87.65	121.15 ± 59.46	142.72 ± 71.48^a^	223.06 ± 107.52^b^^,^^c^
AV45-PET Centiloid values	12.31 ± 31.61	−0.71 ± 16.50	7.47 ± 26.92^a^	39.13 ± 38.83^b^^,^^c^
Centiloid ≤ 10 (n%)	903 (67.1%)	410 (87.6%)	400 (72.6%)^a^	93 (28.4%)^b^^,^^c^
10 < Centiloid ≤ 30 (n%)	110 (8.2%)	27 (5.8%)	49 (8.9%)	34 (10.4%)^b^
Centiloid > 30 (n%)	333 (24.7%)	31 (6.6%)	102 (18.5%)^a^	200 (61.2%)^b^^,^^c^
MK6240-PET [n]	604	207	233	144
Temporal meta-ROI tau SUVR	1.34 ± 0.64	1.02 ± 0.18	1.18 ± 0.43^a^	1.91 ± 0.82^b^^,^^c^

Data are shown as means ± SD or n (%). Group comparisons are performed using ANOVA or Chi-squared test based on the data type. Bonferroni correction is applied to all multiple comparisons.

Abbreviations: ADL, Activities of Daily Living; *APOE*, apolipoprotein E; AV45, 18F-florbetapir; CN, cognitively normal; GFAP, glial fibrillary acidic protein; MCI, mild cognitive impairment; MoCA-B, Montreal Cognitive Assessment-Basic; PET, positron emission tomography; SUVR, standardized uptake value ratio.

a Different from CN and MCI.

b Different from CN and dementia.

c Different from MCI and dementia.

### Plasma P-tau217 and GFAP for distinguishing Centiloid scales

**Fig. 1:**
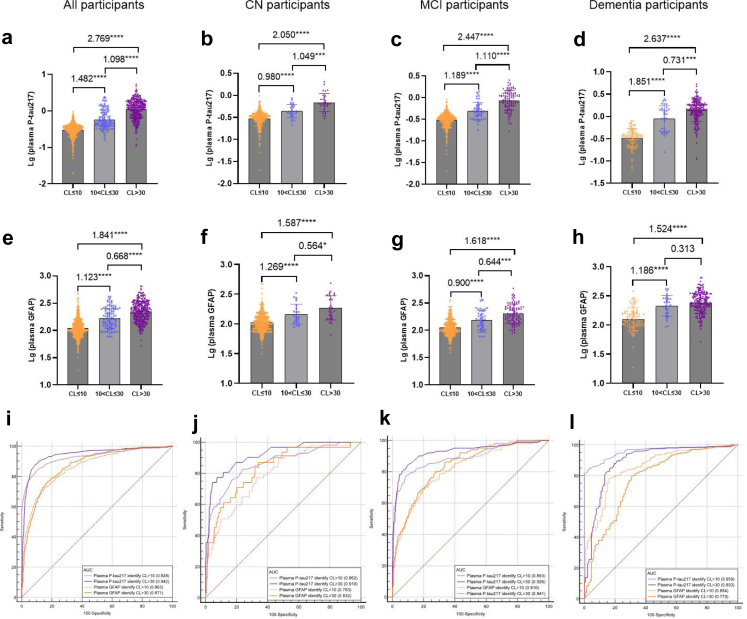
**Changes in plasma P-tau217 and GFAP across different Centiloid scales and their abilities in identifying Centiloid > 10 and Centiloid > 30.** a–h, Differences in plasma P-tau217 and GFAP among groups with varying Centiloid levels. i–l, ROC analyses for plasma P-tau217 and GFAP in identifying individuals with Centiloid > 10 and Centiloid > 30. Analyses are carried out in all participants and those at different cognitive stages, respectively. Plasma P-tau217 and GFAP are log-transformed. Group comparisons are performed using the ANOVA with Bonferroni correction. Effect sizes are reported as Cohen's d to reflect differences between groups. ∗∗∗∗P < 0.0001, ∗∗∗P < 0.001, ∗∗P < 0.005. AUC, area under the curve; CL, Centiloid; GFAP, glial fibrillary acidic protein.

Supplementary Table S1 demonstrated the significant interaction effects of Centiloid scales and cognitive stages on plasma P-tau217, plasma GFAP, and MK6240-SUVR (temporal meta-ROI) levels. Fig. 1a–h shows lg-transformed plasma P-tau217 and GFAP levels across different CL scales. Plasma P-tau217 and GFAP levels significantly increased from the CL ≤ 10 subgroup to the 10 < CL ≤ 30 and CL > 30 subgroups. This trend was consistent across cognitive stages, except for plasma GFAP levels between 10 < CL ≤ 30 and CL > 30 in dementia patients. Supplementary Figure S1 shows MK6240-SUVR (temporal meta-ROI) across CL scales. While individuals with CL > 30 exhibit significantly higher MK6240-SUVR regardless of cognitive stage, a notable increase within the 10 < CL ≤ 30 range is observed only in dementia patients. Fig. 1i–l and Supplementary Table S2 presents the results for plasma biomarkers in distinguishing individuals with CL > 10 and CL > 30. Although not statistically significant, plasma P-tau217 and GFAP exhibited a trend of higher AUCs for identifying individuals with CL > 30 (0.942 and 0.871, respectively) compared to CL > 10 (0.928 and 0.863, respectively) in all participants, as well as in the CN and MCI subgroups. Conversely, in participants with dementia, P-tau217 and GFAP demonstrated significantly higher AUCs for identifying individuals with CL > 10 (0.959 and 0.854, respectively) compared to CL > 30 (0.893 and 0.779, respectively). Detailed sensitivity and specificity metrics for the accuracy of plasma P-tau217 are presented in Supplementary Table S3.

### Dynamic trajectories of plasma P-tau217 and GFAP with Centiloid values

**Fig. 2:**
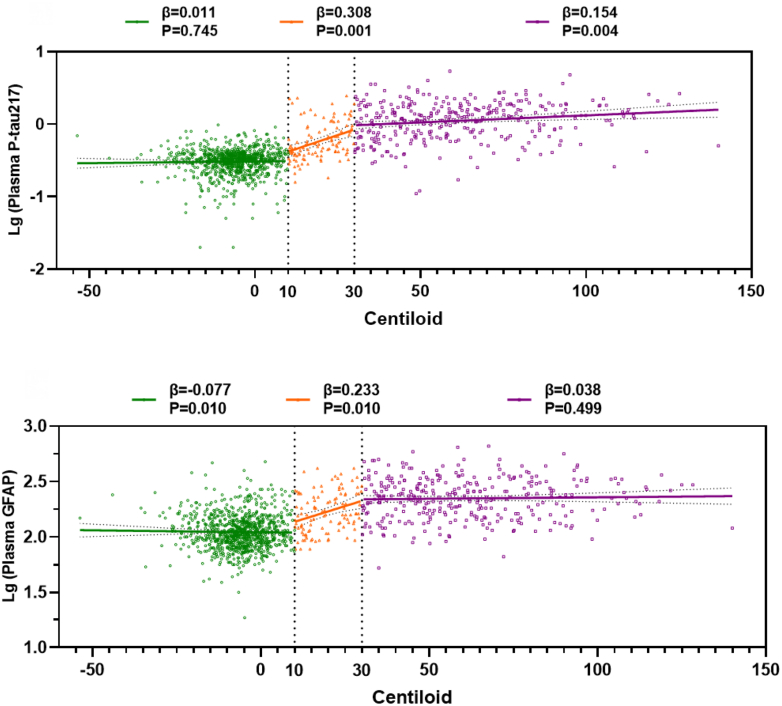
**Evolution of plasma P-tau217 and GFAP in response to increasing Centiloid (CL) values across various scales.** Vertical dashed lines represent CL values of 10 and 30. The β and P values are derived from the linear regression models across individuals with CL ≤ 10, 10 < CL ≤ 30, and CL > 30, adjusted for gender, age, and *APOE* ε4 genotype. Plasma P-tau217 and GFAP are log-transformed.

The progression of plasma P-tau217 and GFAP levels in relation to increasing CL values across various CL scales is depicted in Fig. 2. For CL ≤ 10, plasma P-tau217 levels remained low and unrelated to CL increases, while plasma GFAP levels exhibited a slight decrease (β = −0.077, P = 0.010). In the range of 10< CL ≤ 30, both plasma P-tau217 (β = 0.308, P = 0.001) and GFAP levels (β = 0.233, P = 0.010) demonstrated a significant positive association with CL values. For CL > 30, higher CL values resulted in a slight but significant increase in plasma P-tau217 (β = 0.154, P = 0.004), whereas GFAP levels did not show a significant increase. We also examined the progression of MK6240 SUVR (temporal meta-ROI) in relation to increasing CL values (see Supplementary Figure S2). For both the CL ≤ 10 and 10 < CL ≤ 30 ranges, MK6240 SUVR did not increase with CL values. For CL > 30, there was a significant link between higher CL values and increased MK6240 SUVR (β = 0.162, P = 0.020).

### Sequential mediation of plasma GFAP and P-tau217 between amyloid and Tau

**Fig. 3:**
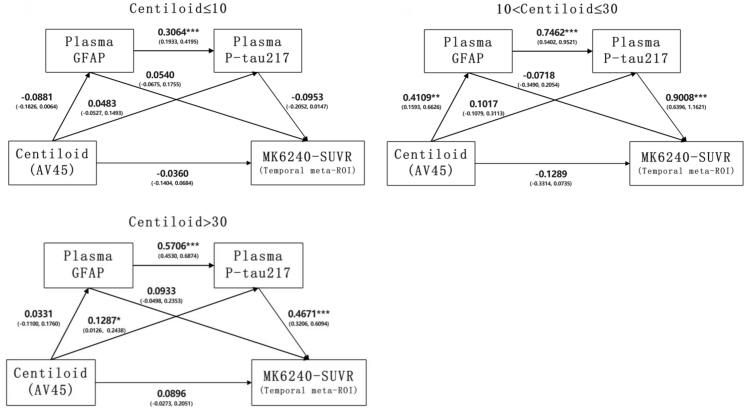
**Sequential mediating effects of plasma GFAP and P-tau217 between Centiloid (CL) values and MK6240 SUVR (temporal meta-ROI) across various CL scales.** Standardized coefficients (95% confidence intervals) are derived from dual mediation models with CL as the independent variable, plasma GFAP and P-tau217 levels as sequential mediators, and MK6240-SUVR (temporal meta-ROI) as the outcome variable. Plasma P-tau217, GFAP, and MK6240-SUVR are all log-transformed. Gender, age, and *APOE* ε4 genotype are included as covariates. ∗∗∗P < 0.0001, ∗∗P < 0.001, ∗P < 0.05.

Fig. 3 illustrates the mediating effects of plasma GFAP and P-tau217 on the link between CL values and MK6240-SUVRs. In the CL < 10 group, higher plasma GFAP levels significantly increased P-tau217 levels (β = 0.3064), but higher CL values did not affect P-tau217, and no link was found between P-tau217 and MK6240-SUVR. In the 10 < CL < 30 group, higher CL values significantly elevated GFAP levels (β = 0.4109), which in turn significantly increased P-tau217 (β = 0.7462), ultimately resulting in a notable rise in MK6240-SUVR (β = 0.9008). When CL exceeds 30, elevated CL values positively affected P-tau217 (β = 0.1287) but not GFAP levels, while an increase in plasma GFAP results in a higher level of P-tau217 (β = 0.5706). The positive effect of plasma P-tau217 on MK6240-SUVR (β = 0.4671) were significant but weaker than in the 10 < CL < 30 group. Across the CL subgroups, no significant direct effects of higher CL values or plasma GFAP on MK6240-SUVR were observed.

### Associations of plasma P-tau217 and GFAP with cortical thickness and cognitive decline

**Fig. 4:**
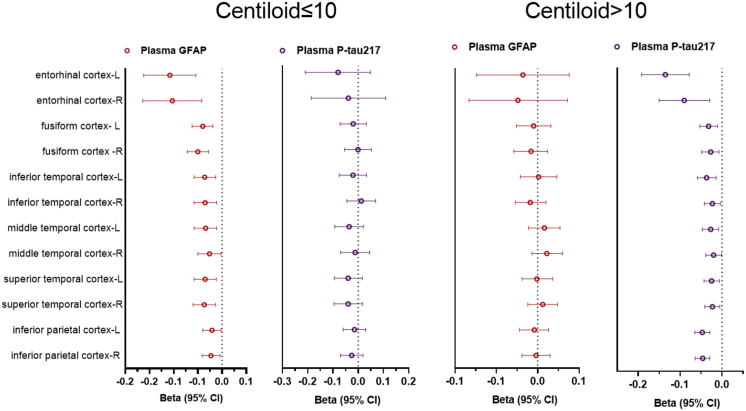
**Associations between plasma P-tau217 and GFAP with cortical thickness in individuals with different Centiloid scales.** Plasma P-tau217 and GFAP levels are z-scored. Beta coefficients (95% CI) and P values are derived from linear regression models, adjusted for gender, age, *APOE* ε4 genotype, and years of education.

**Fig. 5:**
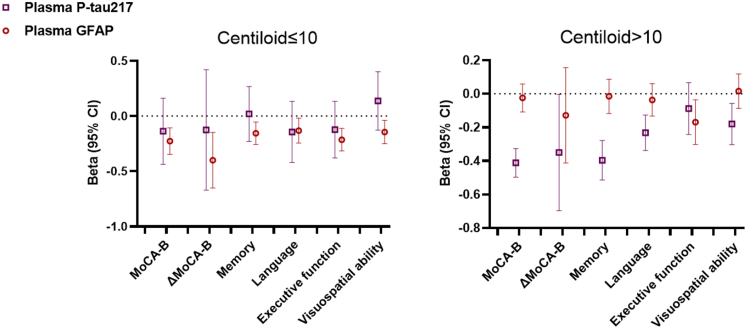
**Associations between plasma P-tau217 and GFAP with cognitive decline in individuals with different Centiloid scales.** Plasma P-tau217 and GFAP levels are z-scored. Beta coefficients (95% CI) and P values represent overall results from linear regression models adjusted for gender, age, APOE ε4 genotype, and years of education. Memory is calculated as the average z-scores of Auditory Verbal Learning Test delayed recall and Brief Visuospatial Memory Test-Revised delayed recall. Language is calculated as the average z-scores of Boston Naming Test and Animal Verbal Fluency Test. Executive function is calculated as the average z-scores of Shape Trail Test Parts A and B. Visuospatial function is calculated as the average z-scores of Silhouette Test and Judgment of Line Orientation. ΔMoCA-B is derived from the annual rate of change in MoCA-B.

Z-scored plasma concentrations of P-tau217 and GFAP were concurrently used to predict cortical atrophy and cognitive decline in groups with CL ≤ 10 and CL > 10 (see Fig. 4). Elevated plasma GFAP was significantly correlated with reduced cortical thickness in the bilateral cerebral cortices, encompassing the entorhinal, inferior temporal, middle temporal, superior temporal, inferior parietal, and fusiform regions, among individuals with CL ≤ 10, but not for those with CL > 10. Conversely, plasma P-tau217 showed no correlation with cortical thickness in the CL ≤ 10 group, but had a significant negative correlation with cortical thickness in the CL > 10 group. The corresponding Beta (95% confidence limits) and P value are presented in Supplementary Table S4. Fig. 5 shows the associations of plasma P-tau217 and GFAP with cognitive decline. In participants with CL ≤ 10, increased plasma GFAP, as opposed to plasma P-tau217, was significantly associated with reduced baseline global cognitive function (MoCA-B), as well as impairments in memory, language, executive function, and visuospatial abilities. Additionally, it was linked to longitudinal global cognitive decline (ΔMoCA-B). Conversely, in participants with a CL > 10, plasma P-tau217, rather than plasma GFAP, showed a significant negative association with MoCA-B, memory, language, visuospatial abilities, and a greater decline in ΔMoCA-B. Notably, an increase in plasma GFAP, but not P-tau217, remained associated with a decline in executive function. The corresponding Beta (95% confidence interval) and P values are presented in Supplementary Table S5.

## Discussion

In this study, our principal findings are as follows: (1) Plasma P-tau217 demonstrated a strong capacity to identify individuals with a CL > 30 within the CN and MCI cohorts, whereas in the dementia cohort, P-tau217 more effectively distinguished individuals with a CL > 10. (2) Increased brain Aβ deposition, indicated by higher CL, resulted in greater tau accumulation, as measured by higher temporal meta-ROI MK6240-SUVR, primarily through a sequential mediation process involving plasma GFAP followed by P-tau217. This effect was particularly pronounced in the CL range of 10 < CL ≤ 30. (3) Elevated levels of plasma GFAP were significantly correlated with brain atrophy and cognitive impairment in the CL ≤ 10 subgroup, whereas elevated P-tau217 levels were associated with brain atrophy and cognitive deficits in non-executive domains among individuals with a CL > 10.

Previous researches have demonstrated that plasma P-tau217 is highly effective in identifying individuals with Aβ-PET positivity.^11^^,^^12^^,^^26^ However, the majority of these studies utilized visual rating or a single SUVR cutpoint to define Aβ+, and many did not examine whether individuals classified as Aβ+ also exhibited cerebral tau pathology. Furthermore, while some studies have suggested that plasma P-tau levels are more closely associated with amyloid rather than tau pathology,^27^^,^^28^ other reports indicating that P-tau217 informs on tau tangle aggregation.^14^^,^^29^ This underscore the need for further investigation into whether the ability of plasma P-tau217 in identifying Aβ+ individuals is related to the concurrent presence of tau pathology. In this study, brain Aβ statuses were categorized into three scales (CL ≤ 10, 10<CL ≤ 30, CL > 30), and the efficacy of plasma P-tau217 in discriminating among CL scales was evaluated across individuals at various cognitive stages. As a result, although P-tau217 increased stepwise from CL ≤ 10 to 10 < CL ≤ 30 to CL > 30, irrespective of cognitive stage, it demonstrated greater efficacy in identifying individuals with elevated MK6240-SUVR, which was evident not only in identifying the CL > 30 group among CN and MCI participants, but also in identifying the CL > 10 group among dementia participants. Thus, we surmise that although plasma P-tau217 rise earlier than the accumulation of NFTs, it offer greater accuracy in identifying Aβ+ individuals with concurrent tau pathology. In any case, according to recent consensus, blood-based biomarker (BBM) tests are anticipated to demonstrate their utility in detecting amyloid pathology as triaging tests, provided they achieve a sensitivity of no less than 90% and a specificity ranging from 75% to 85%.^30^ Our findings, detailed in Supplementary Table S3, indicate that plasma P-tau217 may function as an effective triage test for identifying CL > 30 in populations with MCI (sensitivity, 90%; specificity, 83.5%; cutpoint, 0.43), while for identifying CL > 10 in dementia populations (sensitivity, 90%; specificity, 87.1%; cutpoint, 0.51). To be noted, variations in Aβ+ prevalence and disease progression were observed among different racial and ethnic groups. Specifically, Aβ+ was more prevalent in Whites compared to Asians among individuals with MCI or dementia (57.8% vs 45.4%),^31^ while Aβ+ Asians experienced faster cognitive decline than Aβ+ non-Hispanic Whites among individuals who were cognitively unimpaired or at the MCI stage.^32^ Thus, the utility of plasma P-tau217 in discriminating different CL scales may vary in different racial and ethnic groups. On the other hand, the anti-Aβ clinical trials published to date have predominantly involved White participants with a CL ≥ 30 (5, 6). In addition to the treatment response associated with amyloid clearance, a significant reduction in plasma P-tau217 and GFAP levels, but not in Tau SUVR, was observed.^33^^,^^34^ Considering potential racial and ethnic disparities, as well as our findings indicating that individuals with 10 < CL ≤ 30 are more likely to exhibit elevated plasma P-tau217 and GFAP levels, and even increased brain Tau SUVR in dementia populations, a lower CL threshold for treatment recruitment may be more appropriate for our patient population.

In our study, plasma P-tau217, rather than MK6240-SUVR, exhibited a linear positive correlation with CL values in the 10 < CL ≤ 30 subgroup, confirming an early elevation of plasma P-tau217 during the initial phases of Aβ deposition, which may precede the formation of tau tangles.^14^ In contrast, both MK6240-SUVR and plasma P-tau217 had relatively weak associations with CL values among the CL > 30 scale, indicating Aβ burden alone is insufficient for advancing tau pathology in AD patients. A similar robust relationship between plasma GFAP levels and CL values was observed within the 10 < CL ≤ 30 range, aligning with the previous finding that increased plasma GFAP occurred during the early stage of Aβ deposition.^35^ By far, although the mediating effects of plasma GFAP and P-tau217 between Aβ deposition and tau tangles have been demonstrated individually,^15^^,^^16^ there has been little research concurrently examined the mediating roles of these two plasma biomarkers. In our study, we employed dual mediation models utilizing plasma GFAP and P-tau217 as sequential mediators across varying CL scales. Our findings revealed that, within the scales of 10 < CL ≤ 30 and CL > 30, CL values predominantly facilitated tau pathology through the sequential mediation pathway from plasma GFAP to P-tau217. Moreover, in the 10 < CL ≤ 30 subgroup, the effect size of plasma P-tau217 on MK6240-SUVR was significantly greater than that observed in the CL > 30 subgroup. These results highlight the critical importance of monitoring these blood biomarkers in the early stages of Aβ accumulation to predict AD progression. In additional, beyond targeting Aβ deposition for clearance, early interventions aimed at regulating astrocyte activation and reducing excessive tau phosphorylation in patients with early Aβ deposition may potentially aid in modulating and suppressing the formation of neurofibrillary tangles in the brain.

To more comprehensively assess the independent effects of plasma P-tau217 and GFAP on brain atrophy and cognitive function, these two biomarkers were simultaneously incorporated into our linear regression models within CL ≤ 10 and CL > 10 subgroups, respectively. Our results revealed that elevated plasma GFAP, but not plasma P-tau217 levels in the CL ≤ 10 group were significantly correlated with cortical atrophy, baseline cognitive impairment, and longitudinal cognitive decline. This align with prior research indicating that elevated plasma levels of GFAP, serving as an indicator of astrocyte reactivity, are not exclusive to AD but are broadly correlated with a range of neurodegenerative disorders and forms of dementia.^17^, ^18^, ^19^ In contrast, within the CL > 10 cohort, regression analyses incorporating both biomarkers demonstrated that, while elevated plasma GFAP was significantly correlated with diminished executive function, it was plasma P-tau217, rather than GFAP, that exhibited a strong association with cortical atrophy, global cognitive function, and deficits across various cognitive domains. These findings further suggest that plasma P-tau217 could function not only as an early diagnostic biomarker but also as a specific indicator of neurodegeneration associated with AD. Moreover, the lack of significant impact of plasma GFAP on cortical atrophy and cognitive function within our regression model, which incorporates both P-tau217 and GFAP, may be obscured by the presence of P-tau217, given its stronger association with neurodegeneration in AD. Taking together our dual mediation models, we propose that the previously observed significant impact of plasma GFAP on neurodegeneration and cognitive impairment in AD may primarily be driven by P-tau217 and neurofibrillary tangles in the brain.

Study limitations should be noted. First, due to the single-center design of this study, the findings need to be validated in diverse populations, especially among different racial and ethnic groups. Second, a limited number of participants underwent follow-up assessments of plasma GFAP and P-tau217, so the longitudinal trajectories of these plasma biomarkers in relation to different CL scales, as well as their association with changes in Aβ/Tau, brain atrophy, and cognition, require further confirmation. Third, the mechanisms underlying the higher tau pathology load observed in within the 10 < CL ≤ 30 range remain unclear and warrant further exploration to enhance our understanding in the progression of AD.

In conclusion, this study investigated the progression of plasma GFAP and P-tau217 across varying CL scales and their associations with Aβ/Tau pathology, brain atrophy, and cognitive decline. The findings highlight significant changes in plasma GFAP and P-tau217 during the early stages of brain Aβ accumulation. Moreover, the research demonstrates the variability in the application of these biomarkers for identifying different CL scales across diverse cognitive conditions. As sequential mediators, plasma GFAP and P-tau217 play a crucial role in the transition from Aβ accumulation to tau neurofibrillary tangles. Furthermore, these biomarkers are essential for assessing brain atrophy and cognitive decline under different Aβ conditions. The results provide valuable insights for the clinical understanding and application of plasma biomarkers in Alzheimer's disease.

## Contributors

All authors participated in the collection of the data, had full access to all the data in the study, and reviewed the manuscript for intellectual content. FFP and QHG designed and coordinated the study. LH, FX, and FFP analyzed the data and were responsible for writing and submitting the manuscript. CCH and QH accessed and verified data. LH, FX, CCH, QH, YHG, QHG, and FFP contributed to data interpretation. All authors had final responsibility for the decision to submit for publication.

## Data sharing statement

The data that support the findings of this study are available from the corresponding author upon reasonable request.

## Declaration of interests

The authors declare no competing financial interests.
