# Supplementary material for: Associations of plasma GFAP and P-tau217 with imaging ATN markers and cognitive decline across Centiloid scales
Source: Lancet Reg Health West Pac. 2026 Feb 20;68:101817. doi: 10.1016/j.lanwpc.2026.101817 (PMC12936769; doi:10.1016/j.lanwpc.2026.101817)
Supplement: Supplementary Material [file mmc1.docx]

**Supplementary material**

Supplementary Methods.

Supplementary Figure 1. Changes in MK6240-SUVR (temporal meta-ROI) across different Centiloid scales.

Supplementary Figure 2. Evolution of MK6240 SUVR (temporal meta-ROI) in response to increasing Centiloid (CL) values across various scales.

Supplementary Table 1. Effects of Centiloid scales and Cognitive stages on plasma P-tau217, GFAP, and MK6240-SUVR (temporal meta-ROI).

Supplementary Table 2. Comparative accuracy of plasma P-tau217 and GFAP for the prediction of CL>10 versus CL>30.

Supplementary Table 3. Performances of plasma P-tau217 for predicting individuals with CL>10 and CL>30.

Supplementary Table 4. Effects of plasma P-tau217 and GFAP on predicting cortical thickness in the individuals with different Centiloid scales.

Supplementary Table 5. Effects of plasma P-tau217 and GFAP on predicting cognitive function in the individuals with different Centiloid scales.

**Supplementary Methods**

1. **Standardized neuropsychological tests**

The MoCA-B was utilized to evaluate global cognitive performance^1^. The Activities of Daily Living (ADL) scale was employed to assess overall functional status^2^. Memory was evaluated through composite scores derived from the Auditory Verbal Learning Test (AVLT)^3^ and the Brief Visuospatial Memory Test-Revised (BVMT-R)^4^. Language abilities were assessed using the Boston Naming Test (BNT)^5^ and the Animal Verbal Fluency Test (AFT)^6^. Visuospatial abilities were measured using the Silhouette Test (ST)^7^ and the Judgment of Line Orientation (JLO)^8^. Executive function was assessed with the Shape Trail Test Part A (STT-A) and Part B (STT-B)^9^. All assessments were conducted by trained and certified neuropsychologists to ensure consistency and accuracy in the administration and scoring of the tests.

1. **MRI Image Processing**

The three-dimensional T1-weighted MRI images were processed using the automated analysis pipeline of Freesurfer version 6.0 (http://freesurfer.net/). The preprocessing steps included format conversion, motion correction, intensity inhomogeneity correction, removal of non-brain tissue, and segmentation of gray and white matter.

1. **PET image acquisition and processing**

PET scans using [18F]AV45 were performed 50 minutes after the intravenous administration of approximately 370 MBq (±10%) of [18F]florbetapir, with each scan having a duration of 20 minutes. Tau scans were conducted between 90 and 110 minutes following the intravenous injection of approximately 185 MBq (±10%) of [18F]MK6240. The PET images were reconstructed using a filtered back projection algorithm, incorporating corrections for attenuation, normalization, dead time, photon attenuation, scatter, and random coincidences. Image preprocessing was carried out using Statistical Parametric Mapping12 (SPM12) software (Welcome Trust Centre for Neuroimaging, London, UK; https://www.fil.ion.ucl.ac.uk/spm). Specifically, the PET images were coregistered to the corresponding T1-weighted MRI images. Subsequently, the T1-weighted images were normalized to the standard Montreal Neurological Institute (MNI) space and segmented into gray matter (GM), white matter (WM), and cerebrospinal fluid (CSF). The derived normalization parameters were then applied to the corresponding PET images. The normalized PET and T1-weighted images were smoothed via a Gaussian filter with a full width at half maximum (FWHM) of 8 mm. For the analysis of volumes of interest (VOI), the PET images were subjected to spatial and intensity normalization without smoothing. For the [18F]MK6240-PET data, the meninges were masked using gray matter (GM) segmentation to minimize potential interference from meningeal spillover into adjacent brain regions^10.^

1. **Plasma P-tau217 and GFAP Assays**

Plasma samples were collected from EDTA-treated blood, followed by centrifuged, aliquoted and stored at -80°C. All samples underwent a single freeze-thaw cycle prior to analysis. Based on the Light-initiated chemiluminescent assay (LiCA®), a dual-antibody sandwich technique with two-step incubations was employed for biomarker detection. Initially, the plasma sample was incubated with Chemibeads coated with detection antibodies and biotinylated capture antibodies to form a sandwich immunocomplex. Subsequently, Sensibeads conjugated with streptavidin were added to bind to the biotin on the immunocomplex. Upon excitation by a 680 nm laser, the Sensibeads generated singlet oxygen molecules, which diffused into the Chemibeads, thereby initiating a chemiluminescence reaction. All biomarker measurements were performed by laboratory technicians who were blinded to the clinical data.

Prior to sample testing, we carried out a comprehensive precision profile evaluation of the assays in accordance with the CLSI EP05 - A3 guideline. This study employed an extensive 3×5×5 design to test two levels of quality controls and three patient plasma samples. The results indicated that all biomarkers displayed excellent precision under all tested conditions. The detailed coefficients of variation (CVs) are presented below:

| The coefficients of variation (CVs) for the repeatability, within-laboratory precision, and between-laboratory precision of the LiCA® p-tau 217 assay. | | | | | | | |
| --- | --- | --- | --- | --- | --- | --- | --- |
| Sample | Mean | Repeatability | | Within-Lab  Precision | | Between--Lab  Precision | |
|  | pg/mL | SD | %CV | SD | %CV | SD | %CV |
| plasma 1 | 0.43 | 0.02 | 4.21 | 0.02 | 4.47 | 0.02 | 4.86 |
| plasma 2 | 1.95 | 0.08 | 3.99 | 0.08 | 3.99 | 0.10 | 5.12 |
| plasma 3 | 4.86 | 0.15 | 3.01 | 0.15 | 3.01 | 0.15 | 3.01 |
| QC L | 1 | 0.05 | 4.87 | 0.05 | 4.87 | 0.05 | 4.87 |
| QC H | 4.98 | 0.14 | 2.77 | 0.14 | 2.87 | 0.15 | 2.96 |



| The coefficients of variation (CVs) for the repeatability, within-laboratory precision, and between-laboratory precision of the LiCA® GFAP assay. | | | | | | | |
| --- | --- | --- | --- | --- | --- | --- | --- |
| Sample | Mean | Repeatability | | Within-Lab  Precision | | Between--Lab  Precision | |
|  | pg/mL | SD | %CV | SD | %CV | SD | %CV |
| plasma 1 | 91.31 | 4.22 | 4.63 | 4.38 | 4.80 | 4.47 | 4.90 |
| plasma 2 | 271.86 | 12.30 | 4.52 | 12.30 | 4.52 | 15.57 | 5.73 |
| plasma 3 | 506.92 | 14.80 | 2.92 | 14.80 | 2.92 | 14.82 | 2.92 |
| QC L | 38.91 | 2.70 | 6.93 | 2.70 | 6.93 | 2.71 | 6.96 |
| QC H | 353.91 | 10.00 | 2.83 | 10.13 | 2.86 | 10.13 | 2.86 |

**References**

1. Huang L, Chen KL, Lin BY, et al. Chinese version of Montreal Cognitive Assessment Basic for discrimination among different severities of Alzheimer's disease. *Neuropsychiatr Dis Treat* 2018; **14**: 2133-40.

2. Chen P, Yu ES, Zhang M, Liu WT, Hill R, Katzman R. ADL dependence and medical conditions in Chinese older persons: a population-based survey in Shanghai, China. *J Am Geriatr Soc* 1995; **43**(4): 378-83.

3. Zhao Q, Guo Q, Liang X, et al. Auditory Verbal Learning Test is Superior to Rey-Osterrieth Complex Figure Memory for Predicting Mild Cognitive Impairment to Alzheimer's Disease. *Curr Alzheimer Res* 2015; **12**(6): 520-6.

4. Benedict RHB, Groninger, L., Schretlen, D., Dobraski, M., & Shpritz, B. Revision of the brief visuospatial memory test: Studies of normal performance, reliability, and, validity. *Psychological Assessment* 1996; **8**(2): 145-53.

5. Guo Q.H. HZ, Shi W.X., Sun Y.M., Lv C.Z. Boston naming test using by Chinese elderly, patient with mild cognitive impairment and Alzheimer’s dementia. *Journal of Chinese Mental Health* 2006; **20**: 81-5.

6. Zhao Q, Guo Q, Hong Z. Clustering and switching during a semantic verbal fluency test contribute to differential diagnosis of cognitive impairment. *Neurosci Bull* 2013; **29**(1): 75-82.

7. Huang L, Chen KL, Lin BY, et al. An abbreviated version of Silhouettes test: a brief validated mild cognitive impairment screening tool. *Int Psychogeriatr* 2019; **31**(6): 849-56.

8. Qualls CE, Bliwise NG, Stringer AY. Short forms of the Benton Judgment of Line Orientation Test: development and psychometric properties. *Arch Clin Neuropsychol* 2000; **15**(2): 159-63.

9. Zhao Q, Guo Q, Li F, Zhou Y, Wang B, Hong Z. The Shape Trail Test: application of a new variant of the Trail making test. *PLoS One* 2013; **8**(2): e57333.

10. Betthauser TJ, Cody KA, Zammit MD, et al. In Vivo Characterization and Quantification of Neurofibrillary Tau PET Radioligand (18)F-MK-6240 in Humans from Alzheimer Disease Dementia to Young Controls. *J Nucl Med* 2019; **60**(1): 93-9.


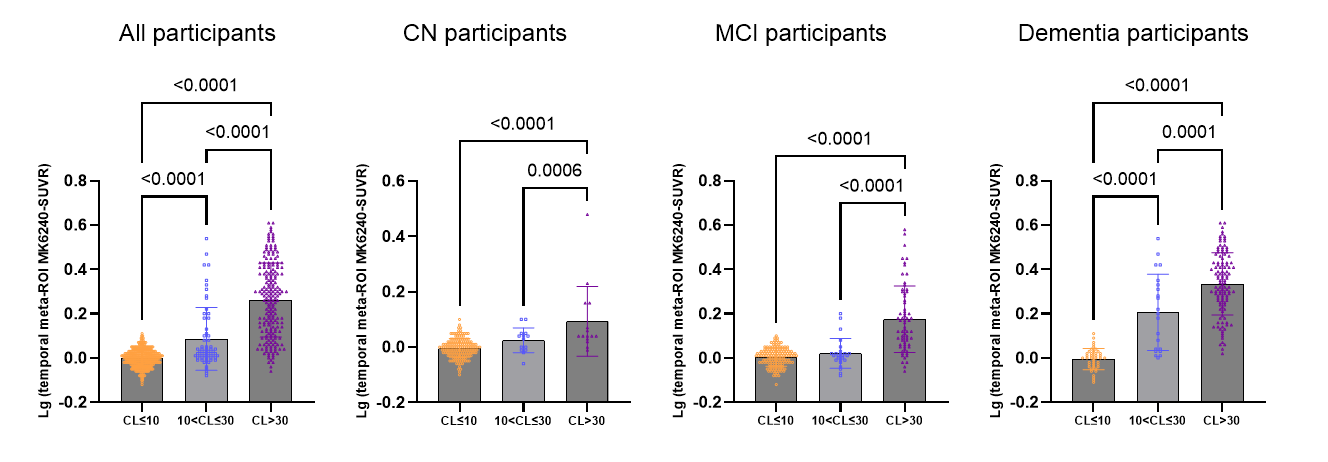
**Supplementary Figure 1. Changes in MK6240-SUVR (temporal meta-ROI) across different Centiloid scales.** Group comparisons are performed using the ANOVA with Bonferroni correction, in all the participants and participants with different cognitive stages, respectively. MK6240-SUVRs (temporal meta-ROI) are log-transformed.


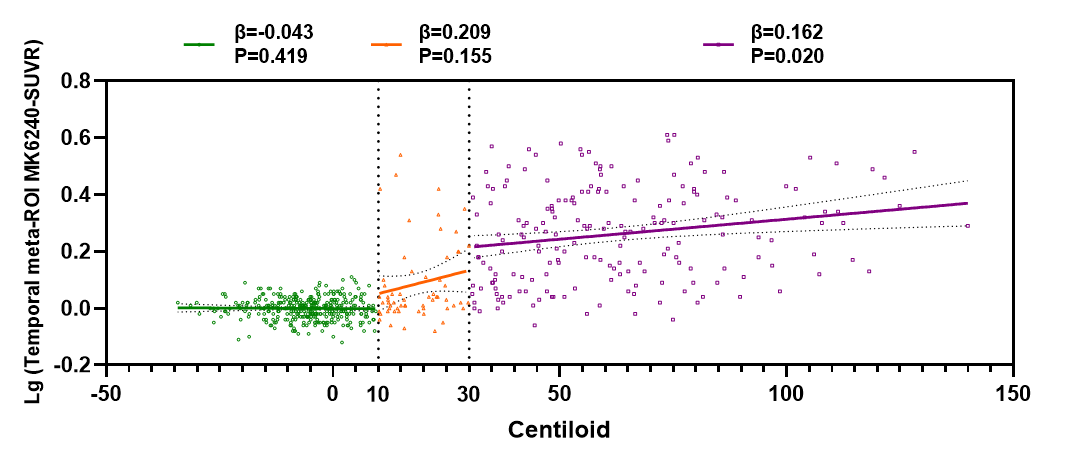
**Supplementary Figure 2. Evolution of MK6240 SUVR (temporal meta-ROI) in response to increasing Centiloid (CL) values across various scales.** Vertical dashed lines represent CL values of 10 and 30. The β and P values are derived from the linear regression models across individuals with CL≤10, 10<CL≤30, and CL>30, adjusted for gender, age, and *APOE* ε4 genotype. MK6240-SUVRs (temporal meta-ROI) are log-transformed.

| **Supplementary Table 1. Effects of Centiloid scales and Cognitive stages on plasma P-tau217, GFAP, and MK6240-SUVR (temporal meta-ROI).** | | | | |
| --- | --- | --- | --- | --- |
| **Index** | **df** | **Mean Square** | **F** | **P** |
| **Lg (plasma P-tau217)** | | | | |
| Corrected Model | 12 | 7.046 | 181.0 | <0.001 |
| Intercept | 1 | 1.422 | 36.5 | <0.001 |
| Centiloid scales (CL≤10, 10＜CL≤30, CL＞30) | 2 | 14.254 | 366.3 | <0.001 |
| Cognitive stages (CN, MCI, Dementia) | 2 | 2.204 | 56.6 | <0.001 |
| Centiloid scales*Cognitive stages | 4 | 0.535 | 13.7 | <0.001 |
| **Lg (plasma GFAP)** | | | | |
| Corrected Model | 12 | 2.458 | 107.3 | <0.001 |
| Intercept | 1 | 34.452 | 1503.8 | <0.001 |
| Centiloid scales (CL≤10, 10＜CL≤30, CL＞30) | 2 | 3.368 | 147.0 | <0.001 |
| Cognitive stages (CN, MCI, Dementia) | 2 | 0.663 | 28.9 | <0.001 |
| Centiloid scales*Cognitive stages | 4 | 0.058 | 2.5 | 0.039 |
| **Lg (temporal meta-ROI MK6240-SUVR)** | | | | |
| Corrected Model | 12 | 0.888 | 102.8 | <0.001 |
| Intercept | 1 | 0.214 | 24.8 | <0.001 |
| Centiloid scales (CL≤10, 10＜CL≤30, CL＞30) | 2 | 1.336 | 154.7 | <0.001 |
| Cognitive stages (CN, MCI, Dementia) | 2 | 0.538 | 62.2 | <0.001 |
| Centiloid scales*Cognitive stages | 4 | 0.195 | 22.6 | <0.001 |
| Statistical tests are calculated using general linear models. CL, centiloid; CN, cognitively normal; MCI, mild cognitive impairment. | | | | |

| **Supplementary Table 2. Comparative accuracy of plasma P-tau217 and GFAP for the prediction of CL>10 versus CL>30.** | | | | | | | | |
| --- | --- | --- | --- | --- | --- | --- | --- | --- |
| **Index** | **All participants**  **(n=1346)** | | **CN participants**  **(n=468)** | | **MCI participants**  **(n=551)** | | **Dementia participants**  **(n=327)** | |
|  | **AUC**  **(95% CL)** | **P** | **AUC**  **(95% CL)** | **P** | **AUC**  **(95% CL)** | **P** | **AUC**  **(95% CL)** | **P** |
| **Plasma P-tau217** | | | | | | | | |
| Identifying CL＞10 | 0.928  (0.913 - 0.941) | 0.2412 | 0.862  (0.827 - 0.892) | 0.1438 | 0.893  (0.864 - 0.917) | 0.1805 | 0.959  (0.931 - 0.977) | 0.0035 |
| Identifying CL＞30 | 0.942  (0.928 - 0.954) |  | 0.919  (0.890 - 0.942) |  | 0.926  (0.901 - 0.947) |  | 0.893  (0.855 - 0.925) |  |
| **Plasma GFAP** | | | | | | | | |
| Identifying CL＞10 | 0.863  (0.843 - 0.881) | 0.5972 | 0.793  (0.754 - 0.829) | 0.4373 | 0.816  (0.781 - 0.847) | 0.3808 | 0.854  (0.811 - 0.891) | 0.0339 |
| Identifying CL＞30 | 0.871  (0.852 - 0.888) |  | 0.832  (0.795 - 0.865) |  | 0.841  (0.808 - 0.871) |  | 0.779  (0.730 - 0.823) |  |
| Differences between various AUCs were compared using Delong test. AUC, area under the ROC curve; CL, centiloid; CN, cognitively normal; MCI, mild cognitive impairment. | | | | | | | | |

| **Supplementary Table 3. Performances of plasma P-tau217 for predicting individuals with CL>10 and CL>30** | | | | | | | |
| --- | --- | --- | --- | --- | --- | --- | --- |
| **Index** | **AUC (95% CL)** | **Sensitivity (%)** | **Specificity (%)** | **Cut point** | **Fixed Sensitivity (%)** | **Estimated Specificity (%)** | **Cut point** |
| **Predicting CL>10** | | | | | | | |
| All participants (n=1346) | 0.928 (0.913 - 0.941) | 83.52 | 90.37 | >0.45 | 90.00 | 78.37 | >0.38 |
| CN participants (n=468) | 0.862 (0.827 - 0.892) | 75.86 | 82.93 | >0.39 | 90.00 | 56.93 | >0.32 |
| MCI participants (n=551) | 0.893 (0.864 - 0.917) | 77.48 | 91.25 | >0.45 | 90.00 | 48.72 | >0.31 |
| Dementia participants (n=327) | 0.959 (0.931 - 0.977) | 84.62 | 96.77 | >0.69 | 90.00 | 87.10 | >0.51 |
| **Predicting CL>30** | | | | | | | |
| All participants (n=1346) | 0.942 (0.928 - 0.954) | 87.69 | 91.02 | >0.53 | 90.00 | 88.51 | >0.49 |
| CN participants (n=468) | 0.919 (0.890 - 0.942) | 77.42 | 92.45 | >0.49 | 90.00 | 73.11 | >0.37 |
| MCI participants (n=551) | 0.926 (0.901 - 0.947) | 84.31 | 90.20 | >0.49 | 90.00 | 83.25 | >0.43 |
| Dementia participants (n=327) | 0.893 (0.855 - 0.925) | 88.00 | 80.31 | >0.69 | 90.00 | 77.17 | >0.61 |
| All values were calculated by receiver operating characteristic (ROC) analyses. AUC, area under the ROC curve; CL, centiloid; CN, cognitively normal; MCI, mild cognitive impairment. | | | | | | | |

| **Supplementary Table 4. Effects of plasma P-tau217 and GFAP on predicting cortical thickness in the individuals with different Centiloid scales.** | | | | | | | | | | | |
| --- | --- | --- | --- | --- | --- | --- | --- | --- | --- | --- | --- |
| **Cortical thickness** | **Participants with CL≤10** | | | | | | **Participants with CL>10** | | | | |
|  | **Plasma GFAP** | | | **Plasma P-tau217** | | | **Plasma GFAP** | | | **Plasma P-tau217** | |
|  | **Beta (95%CI)** | **P** | | **Beta (95%CI)** | **P** | | **Beta (95%CI)** | **P** | | **Beta (95%CI)** | **P** |
| Entorhinal cortex (L) | -0.108(-0.162, -0.054) | <0.001 | -0.080(-0.210, 0.049) | | 0.223 | -0.018(-0.074, 0.038) | | 0.526 | -0.135(-0.192, -0.078) | | <0.001 |
| Entorhinal cortex (R) | -0.103(-0.164, -0.042) | 0.001 | -0.039(-0.186, 0.109) | | 0.606 | -0.024(-0.083, 0.036) | | 0.432 | -0.090(-0.150, -0.029) | | 0.004 |
| Fusiform cortex (L) | -0.040(-0.062, -0.019) | <0.001 | -0.020(-0.072, 0.032) | | 0.444 | -0.005(-0.026, 0.016) | | 0.626 | -0.032(-0.053, -0.011) | | 0.003 |
| Fusiform cortex (R) | -0.050(-0.072, -0.028) | <0.001 | -0.001(-0.054, 0.052) | | 0.970 | -0.008(-0.029, 0.012) | | 0.424 | -0.027(-0.048, -0.007) | | 0.010 |
| inferior temporal cortex (L) | -0.036(-0.058, -0.013) | 0.002 | -0.021(-0.075, 0.033) | | 0.447 | 0.001(-0.021, 0.023) | | 0.938 | -0.037(-0.059, 0.014) | | 0.001 |
| inferior temporal cortex (R) | -0.035(-0.058, -0.011) | 0.004 | 0.012(-0.045, 0.069) | | 0.677 | -0.009(-0.027, 0.010) | | 0.371 | -0.023(0.042, -0.004) | | 0.017 |
| middle temporal cortex (L) | -0.034(-0.058, -0.011) | 0.005 | -0.036(-0.093, 0.021) | | 0.216 | 0.008(-0.011, 0.027) | | 0.386 | -0.027(-0.047, -0.008) | | 0.005 |
| middle temporal cortex (R) | -0.026(-0.05, -0.002) | 0.031 | -0.012(-0.070, 0.045) | | 0.675 | 0.011(-0.007, 0.030) | | 0.238 | -0.020(-0.039, -0.001) | | 0.037 |
| superior temporal cortex (L) | -0.035(-0.058, -0.012) | 0.003 | -0.040(-0.095, 0.016) | | 0.161 | -0.001(-0.019, 0.018) | | 0.930 | -0.025(-0.043, -0.006) | | 0.011 |
| superior temporal cortex (R) | -0.037(-0.060,-0.013) | 0.002 | -0.04 (-0.096, 0.017) | | 0.166 | 0.006(-0.012, 0.024) | | 0.494 | -0.023(-0.041, -0.005) | | 0.012 |
| inferior parietal cortex (L) | -0.021(-0.040, -0.002) | 0.028 | -0.015 (-0.06, 0.030) | | 0.516 | -0.004(-0.022, 0.013) | | 0.614 | -0.047(-0.065, -0.029) | | <0.001 |
| inferior parietal cortex (R) | -0.023(-0.041, -0.004) | 0.018 | -0.026(-0.071, 0.019) | | 0.262 | -0.002(-0.019, 0.015) | | 0.795 | -0.046(-0.064, -0.029) | | <0.001 |
| Beta coefficients (95% CI) and P values are derived from overall tests in linear regressions models, adjusted for gender, age, *APOE* ε4 genotype, and years of education. Plasma P-tau217 and GFAP are z-scored. | | | | | | | | | | | |

| **Supplementary Table 5. Effects of plasma P-tau217 and GFAP on predicting cognitive function in the individuals with different Centiloid scales.** | | | | | | | | | | | |
| --- | --- | --- | --- | --- | --- | --- | --- | --- | --- | --- | --- |
| **Cognitive function** | **Participants with CL≤10** | | | | | | **Participants with CL>10** | | | | |
|  | **Plasma GFAP** | | | **Plasma P-tau217** | | | **Plasma GFAP** | | | **Plasma P-tau217** | |
|  | **Beta (95%CI)** | **P** | | **Beta (95%CI)** | **P** | | **Beta (95%CI)** | **P** | | **Beta (95%CI)** | **P** |
| MoCA-B | -0.227(-0.346, -0.107) | <0.001 | -0.137(-0.435, 0.161) | | 0.366 | -0.024(-0.108, 0.059) | | 0.564 | -0.411(-0.497, -0.326) | | <0.001 |
| ΔMoCA-B | -0.400(-0.651, -0.148) | 0.002 | -0.126(-0.670, 0.419) | | 0.650 | -0.128(-0.412, 0.156) | | 0.373 | -0.350(-0.697, -0.003) | | 0.048 |
| ^a^Memory | -0.156(-0.257, -0.055) | 0.003 | 0.019(-0.229, 0.267) | | 0.880 | -0.015(-0.117, 0.087) | | 0.778 | -0.396(-0.514, -0.278) | | <0.001 |
| ^b^Language | -0.132(-0.244, -0.019) | 0.021 | -0.144(-0.420, 0.133) | | 0.309 | -0.036(-0.132, 0.061) | | 0.465 | -0.232(-0.338, -0.126) | | <0.001 |
| ^c^Executive function | -0.214(-0.316, -0.111) | <0.001 | -0.123(-0.379, 0.133) | | 0.347 | -0.169(-0.302, -0.036) | | 0.013 | -0.088(-0.242, 0.066) | | 0.261 |
| ^d^Visuospatial ability | -0.144(-0.251, -0.038) | 0.008 | 0.137(-0.127, 0.400) | | 0.309 | 0.016(-0.087, 0.119) | | 0.761 | -0.180(-0.303, -0.057) | | 0.004 |
| Beta coefficients (95% CI) are for overall tests in linear regressions adjusted for gender, age, APOE ε4 genotype, and years of education. Plasma P-tau217 and GFAP are z-scored. ΔMoCA-B is derived from the annual rate of change in MoCA-B. ^a^Memory is calculated as the average of the z-scores for Auditory Verbal Learning Test delayed recall and Brief Visuospatial Memory Test-Revised delayed recall. ^b^Language is calculated as the average of the z-scores for Boston Naming Test and Animal Verbal Fluency Test. ^c^Executive function is calculated as the average of the negative z-scores for Shape Trail Test Part A and B. ^d^Visuospatial ability is calculated as the average of the z-scores for Silhouette Test and Judgement of Line Orientation. | | | | | | | | | | | |
